# Treatment strategies for smartphone addiction: efficacy study of transcranial direct current stimulation and exergaming

**DOI:** 10.3389/fpubh.2024.1416976

**Published:** 2024-11-05

**Authors:** Jun Chen, YuQing Jia, YanXia Zhu, Qin Liu, Fan Cheng, Bo Yang, EnMing Zhang

**Affiliations:** ^1^School of Sports Medicine and Rehabilitation, Beijing Sport University, Beijing, China; ^2^The Cardiopulmonary Rehabilitation Center at Taihe Hospital, Shiyan, China; ^3^Imaging Department of Taihe Hospital, Shiyan, China; ^4^Key Laboratory of Exercise Rehabilitation Science of the Ministry of Education, Beijing Sport University, Beijing, China

**Keywords:** smartphone addiction, transcranial direct current stimulation, exergames, event-related potentials, addictive behavior, treatment

## Abstract

**Objective:**

Smartphone addiction is on the rise globally. This study aimed to compare the rehabilitative effects of transcranial direct current stimulation (tDCS) and exergames on smartphone addiction among college students. Additionally, we discussed the central mechanisms through changes in electroencephalography (EEG) to provide clinical insights.

**Methods:**

Thirty-six participants were randomly assigned to three groups: control group, tDCS group (bilateral dorsolateral prefrontal cortex stimulation), and exergame group. The intervention lasted for 4 weeks with twice-weekly sessions. Outcome measures included the Smartphone Addiction Scale - Chinese Version (SAS-C), Iowa Gambling Task (IGT) net scores, and event-related potential (ERP) data collected during the IGT, focusing on P300 and feedback-related negativity (FRN) amplitudes.

**Results:**

All groups showed significant reductions in SAS-C scores post-intervention. Behaviorally, post-intervention scores improved, indicating significant effects of different interventions on participants’ strategy choices. P300 amplitudes increased significantly at outcome electrode sites for all groups, with the most notable increase in tDCS group FC2 and CP1. FRN amplitudes decreased significantly post-intervention in the control and tDCS groups, with significant differences between the two groups.

**Conclusion:**

All three interventions appeared to have alleviating effect on smartphone addiction. After 4 weeks, participants showed improved executive control and decision-making abilities. Specifically, significant effects were observed in the tDCS group, with increased P300 amplitudes in the frontal, parietal, and central regions, as well as FRN amplitudes in the central and frontal regions. This suggested that tDCS enhanced psychological resources and improved inhibition control capabilities.

## 1 Introduction

In contemporary society, smartphones have seamlessly integrated into the fabric of our daily lives, evolving into an indispensable tool. As of 2022, the global count of smartphone users has nearly doubled since 2016, surging from 3.7 billion to an impressive 6.6 billion (1). Noteworthy is China’s distinctive contribution, boasting 986 million smartphone internet users in the first half of 2021 (2). As indicated by a comprehensive meta-analysis, China is among the nations grappling with elevated levels of smartphone addiction (3). About 36.6% of college students were particularly susceptible to smartphone addiction, manifesting in their average daily screen time rising sharply from 3.75 h in 2012 to 5.78 h in 2017 (4). These statistics highlighted the urgent need for prevention and intervention strategies for smartphone addiction.

It is a pity that the current Diagnostic and Statistical Manual of Mental Disorders-5 (DSM-5) does not formally categorize smartphone addiction. Nevertheless, scholars characterize it as an excessive and uncontrollable usage pattern that disrupts or distorts various facets of an individual’s daily existence, including work, academic pursuits, behavior, social interactions, and mental well-being (5–7). Nexus between smartphone addiction and conditions such as anxiety, depression (8), loneliness (9), interpersonal relationships (10), social support (11), familial environment, and aggressive personality traits (12) has been identified. Additionally, it may lead to physical issues such as neck and shoulder pain (13) traffic accidents (14), sensory problems (15) as well as cognitive impairments in executive functions according to present studies.

Presently, mainstream treatments for smartphone addiction predominantly encompass Cognitive Behavioral Therapy (CBT), motivational interventions, and mindfulness-based cognitive-behavioral therapy (16). While these methods provide therapeutic benefits, their effectiveness is limited by environmental influences, hardware constraints, and adaptability. The accessibility of treatment resources for individuals with smartphone addiction continues to be a challenge.

Transcranial Direct Current Stimulation (tDCS) emerges as a promising non-invasive neurostimulation method. It involves the placement of electrodes on the scalp to deliver a subtle direct current to the brain, modulating neuronal excitability and altering neural activity patterns. Extensively employed in addiction treatment, tDCS has demonstrated positive outcomes in addressing cravings, dependency (17), cognitive test errors, and addictive behaviors (18).

Concurrently, exergaming, an amalgamation of dynamic posture control, sensory stimulation, and cognitive engagement, offers a universal, interactive, and aerobic exercise solution. Game elements in exergaming temporarily elevate dopamine release in the striatum (19), enhancing cognitive control functions and inducing changes in brain-derived neurotrophic factor (BDNF) release (20). Previous studies have proved that addiction has been linked to impaired Executive Function (EF), including inhibitory control (IC), cognitive flexibility, and working memory (21). Cognitive research suggests that individuals with addiction encounter challenges in inhibition and decision-making (22). In light of this, exercise has been shown to significantly enhance executive function (EF) (22, 23). Both acute and aerobic exercises have been found to exert positive effects on inhibitory control (24, 25), while moderate-intensity exercise can improve working memory (26). Consequently, exergames may emerge as an effective intervention for augmenting inhibition control and mitigating smartphone addiction among college students.

In summary, building upon existing research and literatures, this paper posited that both transcranial direct current stimulation and exergaming hold the potential to significantly mitigate smartphone addiction severity, reinforce inhibitory control, and restore executive functions in individuals with smartphone addiction. This study aimed to compare the therapeutic effects of various interventions on smartphone addiction and provide a theoretical foundation for clinical practice.

## 2 Materials and methods

### 2.1 Design and sample

This study employed a longitudinal intervention comparative analysis design is employed in this study to compare Chinese university students with smartphone addiction before and after interventions involving transcranial direct current stimulation (tDCS), pseudo-stimulation, and exergames. The objective was to compare the efficacy of different interventions on subjective addiction levels, behavioral outcomes, and neurophysiological indicators.

An *a priori* power analysis (G*Power Version 3.1) indicated that a minimum of 24 participants are needed to obtain an alpha level of 0.05 and a statistical power of 0.45 based on moderate effect size. The sample consists of 102 potential participants screened based on inclusion and exclusion criteria, resulting in a final cohort of 36 participants meeting the experimental requirements. Each group comprises 6 males and 6 females, with ages of 19.7 ± 0.76, 19.5 ± 1.3, and 19.9 ± 1.68 years, and educational durations of 14.9 ± 1.38, 15.0 ± 1.6, and 15.0 ± 1.4 years.

Inclusion criteria include: (a) absence of physiological, psychological, or neurological disorders; (b) no history of substance abuse such as alcohol, cannabis, or caffeine; (c) non-engagement in regular physical activity (at least 3 days/week, 30 min/day, moderately intense planned systematic physical activity for at least three months); (d) Smartphone Addiction Scale–Chinese version (SAS-C) score not less than 40; (e) commitment to participate exclusively in this experiment while maintaining regular daily habits.

### 2.2 Interventions

#### 2.2.1 Transcranial direct current stimulation (tDCS)

The transcranial direct current stimulation group received 2 mA transcranial direct current stimulation twice a week for 20 min each time, lasting for 4 weeks. The stimulation site was the F3 and F4 poles of the 10–20 system, with the right prefrontal cortex as the anode stimulation point and the left prefrontal cortex as the cathode stimulation point (27).

#### 2.2.2 Pseudo-stimulation (Sham)

Pseudo stimulation was used as the control to eliminate the placebo effect. The pseudo-stimulation group followed the same protocol with the transcranial direct current stimulation group. However, the stimulator was only activated at 2 mA for the first and last 30 s of the 20-min session. This protocol was designed to mimic the initial sensation experienced in the active condition while avoiding continuous stimulation, a technique previously validated for its efficacy (28). In addition, the participants in control group will be given health education, explaining the risk factors of mobile phone addiction, the impact on vision, posture, psychology, and preventive measures.

#### 2.2.3 Exergames

The exergames group preheated for 5 min before the intervention, and then performed a cognitive somatosensory game intervention with an intensity of 60–80% VO_2max_ (29). During the intervention, participants were required to avoid obstacles (puddles, rolling logs) while running, virtually cross a single plank bridge, and randomly answer questions twice a week for 20 min each time, lasting for 4 weeks. After exercise, they stretched for 5 min.

### 2.3 Measurement tools

#### 2.3.1 Smartphone addiction scale–Chinese version (SAS-C)

The SAS-C, utilized in this study, demonstrates good internal consistency reliability (Cronbach’s *α* = 0.83) and test–retest reliability (test–retest reliability = 0.89). This scale employs a Likert 6-point scoring system, with scores ranging from 1 to 6 for each item and a total score of 40 as the cut-off point for addiction.

#### 2.3.2 Iowa gambling task (IGT)

**Figure 1:**
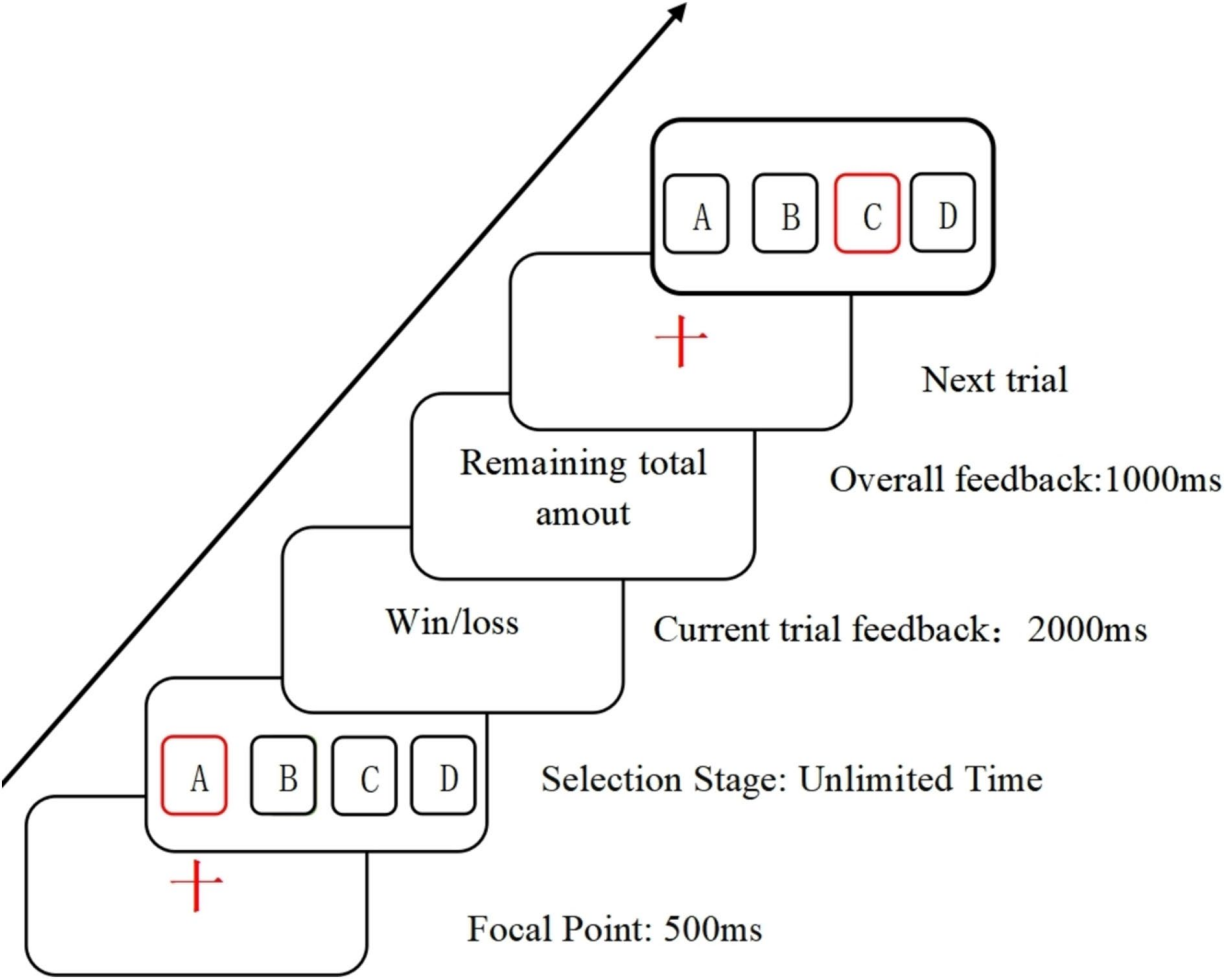
Schematic representation of the Iowa gambling task procedure.

The IGT (Figure 1) involves four cards labeled A, B, C, and D, with two cards being high-risk and the other two low-risk. Participants, starting with a hypothetical initial fund of $2000, need to adjust their card selection strategy based on acquired experience or emotional feedback to achieve a monetary gain. The primary purpose of the Iowa Gambling Task is to calculate the net winning score = (A + C) - (B + D), allowing analysis of decision-making strategies and characteristics in the context of gain-loss scenarios. For each choice made by the participant, the feedback interface will display the number of gains and losses of the selected card and the current cumulative amount of the participant. The entire experiment consists of 6 blocks, each consisting of 10 trials (30). In the Iowa gambling task, a “block” usually refers to a set of strategies or decisions that participants take in the game. In game analysis, a block can represent a group of interconnected decisions or choices that can be viewed as a whole in specific situations.

#### 2.3.3 EEG testing procedure and indicators

The experimental procedure will be explained the experimenter, including the required time and relevant precautions. To ensure the accuracy of experimental data, participants are instructed to relax their entire body, avoid limb movements, keep their mouth closed, and control swallowing, eye movements, and blinking. Subsequently, participants will be asked to sign an informed consent form for the experiment and complete the SAS-C scale as well as the “Transcranial Electrical Stimulation Safety Screening Scale.”

**Figure 2:**
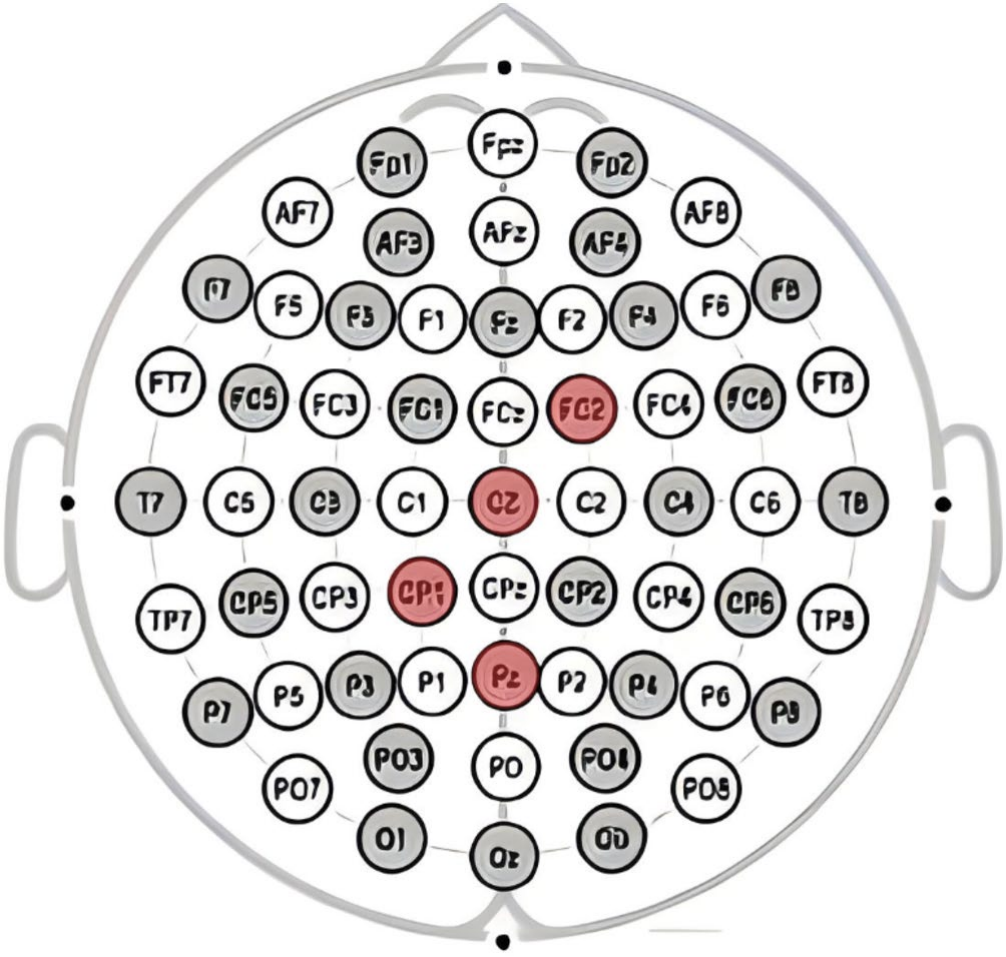
Electrode points Cz, Pz, FC2, and CP1 selected for analyzing the P300 component.

**Figure 3:**
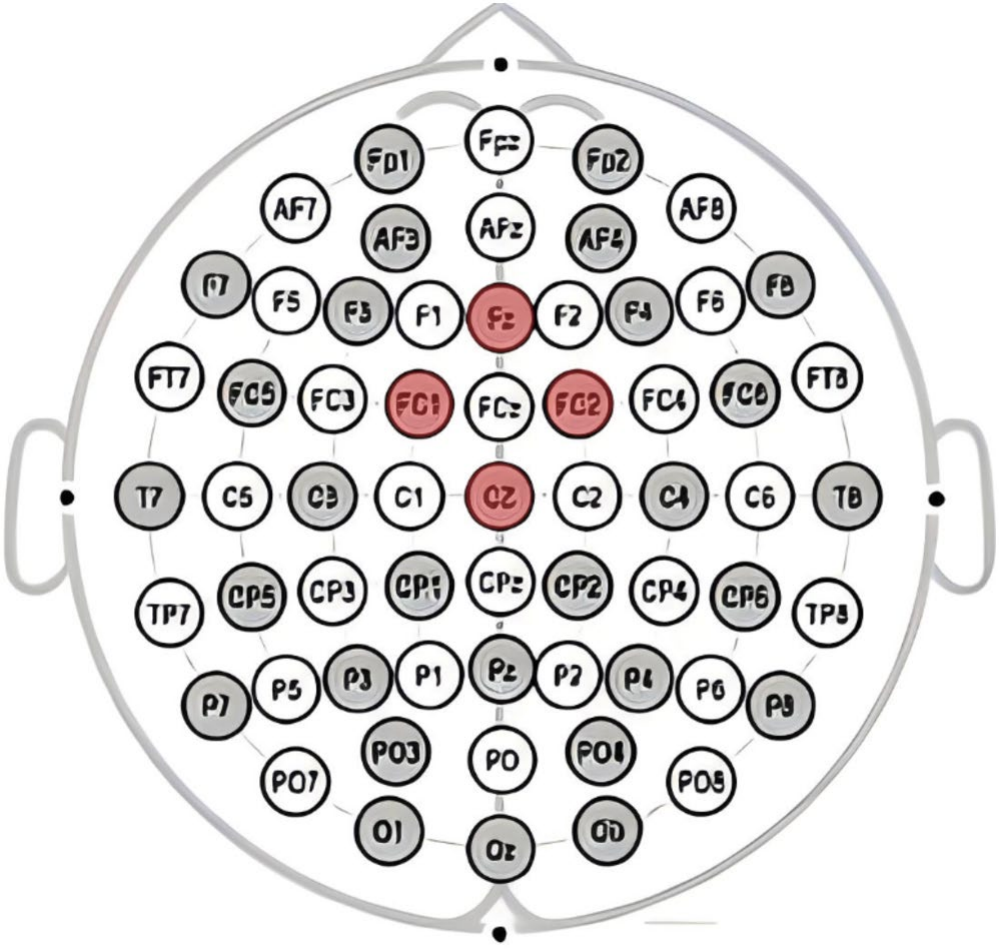
Electrode points Cz, Fz, FC2, and FC1 selected for analyzing the FRN component.

This study utilizeded the NE Neuroelectrics 32-channel electroencephalography (EEG) equipment from Spain for the acquisition of brainwave signals. The system mainly consists of three components: the EEG cap, amplifier, and Scan4.3 signal acquisition and data processing. Electrode placement follows the guidelines of the international 10–20 system, with electrode points Cz, Pz, FC2, and CP1 selected for analyzing the P300 component as shown in Figure 2, and electrode points Cz, Fz, FC2, and FC1 selected for analyzing the FRN component as shown in Figure 3, based on the literature review. In this study, a high-pass filter was set at 0.1 Hz, a notch filter at 50 Hz, and electrode impedance was maintained below 15kΩ.The laboratory temperature was maintained at 25°C, and during the experiment, the lab environment was free of noise, with soft lighting in the room.

### 2.4 Data analysis

Data analysis and processing utilize Excel, Matlab, and SPSS 23.0 software. Behavioral data undergoes statistical analysis using paired-sample t-tests. For EEG data, repeated measures analysis of variance (ANOVA) is employed to analyze main and interaction effects.

### 2.5 Ethical review

All procedures in this study adhere to ethical standards set by relevant institutions and national committees regarding human experimentation, as outlined in the 2000 revised Declaration of Helsinki. The experiment has received ethical approval from the Ethics Review Committee for Sports Science Experiments at Beijing Sport University, with reference number N0: 2022211H. The experiment follows the principles of voluntary participation, confidentiality, justice, and minimal harm. Participants sign informed consent forms, ensuring a comprehensive understanding of the experiment’s purpose, procedures, and potential risks.

## 3 Results

### 3.1 Smartphone addiction scale scores (SAS-C)

The alterations in subjective addiction scores among the three participant groups pre-and post-intervention are delineated in Table 1. Upon intra-group scrutiny, noteworthy changes were observed in both the control group and the transcranial direct current stimulation (tDCS) group (*p* = 0.017 and 0.018, respectively). However, the latter exhibited no statistically significant variance between post-and pre-intervention scores. Inter-group analysis conducted prior to the intervention indicated no significant disparities in SAS-C scores among the control group, tDCS group, and the exergaming group.

**Table 1:** SAS-C scores before and after interventions (M ± SD).

Group	Intervention		*T*	*P*
	Before	After		
Pseudo-stimulation	44.97 ± 3.15	31.71 ± 6.66	7.35	0.02
tDCS	46.25 ± 2.78	36.36 ± 13.44	2.11	0.08
exergame	47.68 ± 4.54	39.56 ± 6.65	2.97	0.02
*F*	1.16	3.38		
*P*	0.45	0.19		

### 3.2 IGT behavioral data results

#### 3.2.1 Pre-intervention strategy selection scores under different blocks

**Figure 4:**
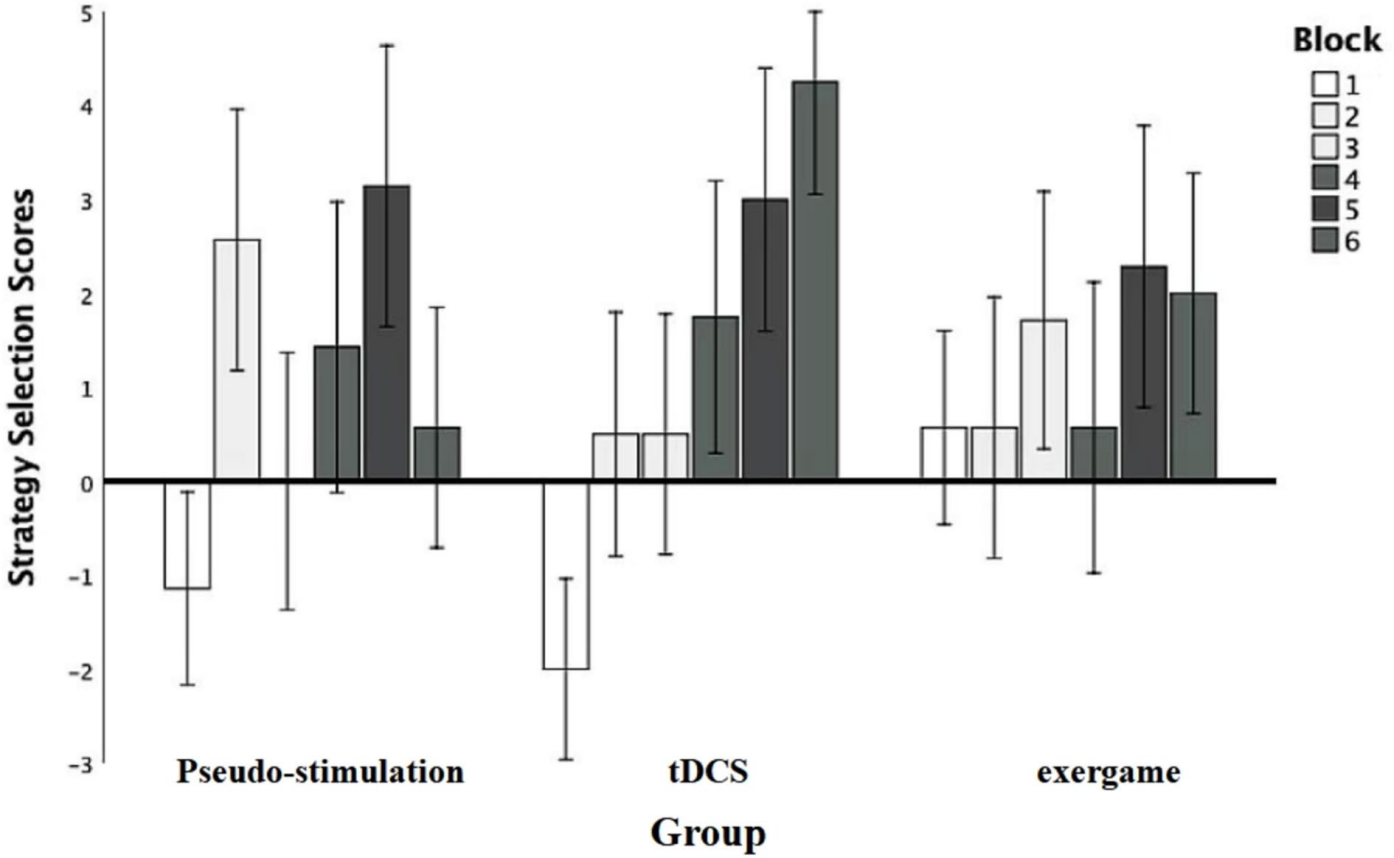
Pre-intervention strategic selection results.

An analysis was initiated with a robust repeated-measures analysis of variance (ANOVA) to meticulously evaluate the impact of diverse blocks on participants’ subjective scores (Figure 4). The results unveiled a compelling main effect *F* (5, 95) = 2.95, *p* = 0.02, statistical power = 0.84, underscoring the substantial influence of module stimulation on the overall performance. Subsequent post-hoc comparisons brought to light significant distinctions: Module 2 scores (2.79 ± 0.83) significantly surpassed those of Module 1 (−0.20 ± 0.91), Module 5 scores (3.85 ± 0.83) significantly outperformed both Module 1 and Module 3 scores (2.85 ± 0.75), and Module 6 scores (4.35 ± 0.75) exhibited significant superiority over Module 1 scores. These disparities were all statistically significant (*p* < 0.05). Nevertheless, the group effect failed to attain significance [*F* (2, 19) = 0.04, *p* = 0.96, statistical power = 0.47], indicating that observed differences among groups could not be solely ascribed to grouping factors.

Moreover, the interaction between grouping and stimulation types did not achieve significance, *F* (10, 95) = 0.95, *p* = 0.50, statistical power = 0.47, elucidating that distinct groups did not manifest noteworthy mutual effects under various stimulation types. These findings accentuate the pivotal role of blocks in shaping participants’ strategic choices, all while highlighting the absence of substantial inter-group variations in response to diverse stimulation types.

#### 3.2.2 Post-intervention strategy selection scores in different groups under various blocks

**Figure 5:**
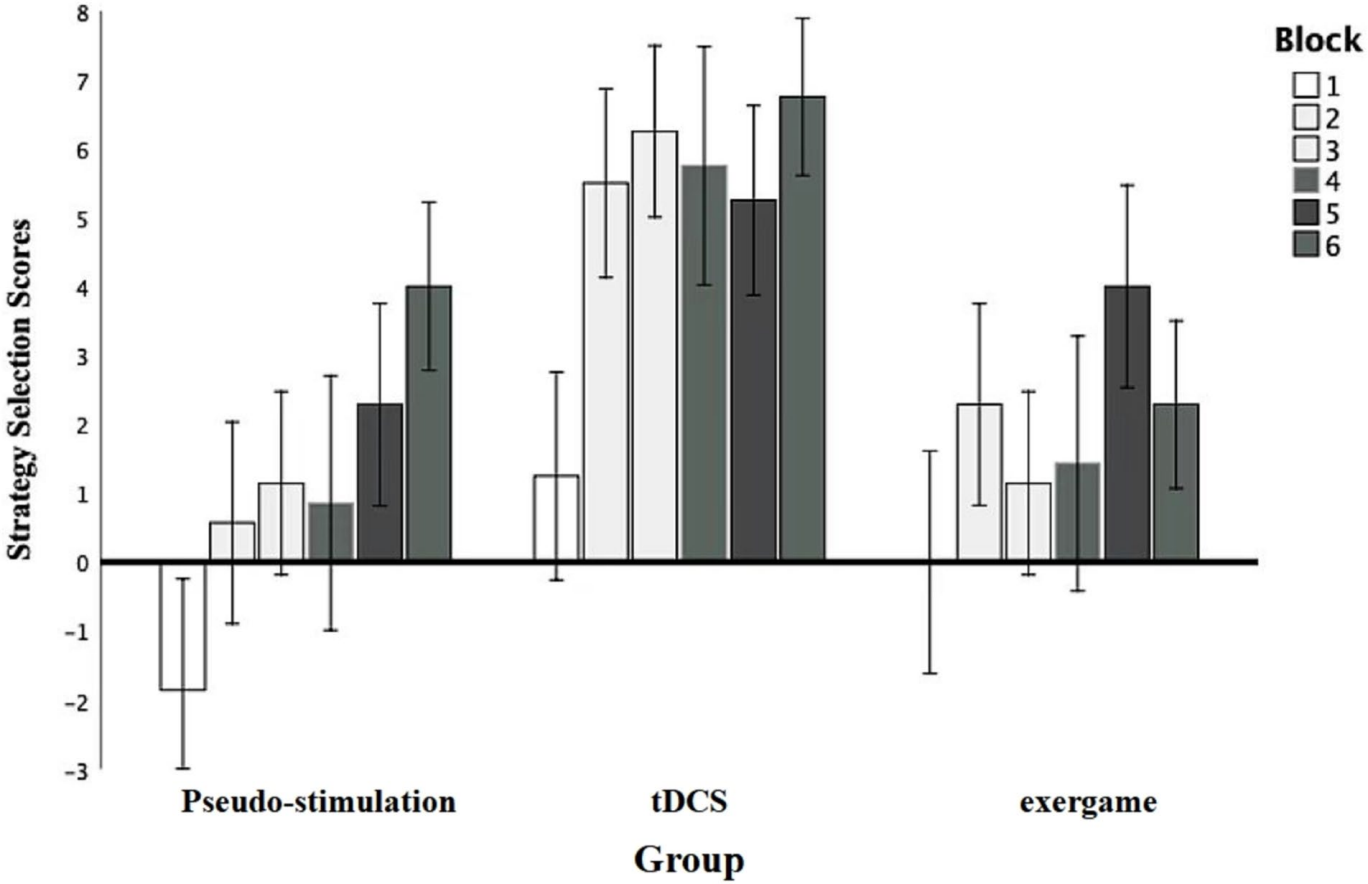
Post-intervention strategic selection results.

An inter-group repeated-measures analysis of variance (ANOVA) was initiated to systematically evaluate the impact of diverse blocks on strategy selection scores (Figure 5). The results unveiled a significant main effect of module stimulation *F* (4.84, 91.86) = 4.61, *p* = 0.00, η2p = 0.20, statistical power = 0.96, underscoring the substantial influence of distinct blocks on strategy selection outcomes, supported by robust statistical power. Further post-hoc comparisons illuminated specific differences. Notably, scores for Modules 2,3,5, and 6 exhibited a statistically significant increase compared to Module 1 (*p* < 0.05), reinforcing the assertion of a substantial impact of individual modules on strategy selection scores.

Additionally, the main effect of grouping was explored. The results indicated a significant main effect among the groups, with *F* (2, 19) = 4.54, *p* = 0.02, η2p = 0.32, and a statistical power of 0.70. Further comparisons revealed that the transcranial direct current stimulation group and the exergame group performed significantly better than the control group, with *p* < 0.05, indicating statistical significance in these differences.

Moreover, the absence of a significant interaction between grouping and stimulus type *F* (10, 95) = 0.95, *p* = 0.50, statistical power = 0.47, suggests that the responses of different groups to various stimulus types did not exhibit a notable mutual influence.

These findings, robustly grounded in statistical theory, accentuate the profound impact of diverse blocks on strategy selection outcomes.

#### 3.2.3 Pre-and post-intervention P300 amplitudes in IGT paradigm

Inter-group repeated-measures analysis of variance (ANOVA) was employed to meticulously scrutinize the disparities in P300 amplitude indicators between pre-and post-intervention assessments across different electrode positions (Table 2).

**Table 2:** P300 amplitudes pre-and post-intervention for participants (M ± SD).

Electrode	Phase	Group				Inter-Group		Intra-Group		Interaction effect	
		Pseudo-stimulation	tDCS	Exergame	Total	*F*	*P*	*F*	*P*	*F*	*P*
Cz	Pre	0.35 ± 0.47	0.52 ± 0.44	−1.64 ± 0.47	−0.26 ± 0.27	9.28	0.00	11.88	0.00	2.95	0.08
	Post	0.92 ± 0.30	1.22 ± 0.28	0.94 ± 0.30	1.03 ± 0.17						
	Total	0.64 ± 0.22	0.87 ± 0.20	−0.35 ± 0.22	0.39 ± 0.12						
Pz	Pre	−0.02 ± 0.34	0.37 ± 0.32	0.24 ± 0.34	0.19 ± 0.19	6.19	0.01	9.09	0.01	3.46	0.05
	Post	0.36 ± 0.19	1.76 ± 0.18	0.37 ± 0.19	0.83 ± 0.11						
	Total	0.17 ± 0.20	1.06 ± 0.19	0.31 ± 0.20	0.51 ± 0.11						
Fc2	Pre	0.25 ± 0.87	−0.14 ± 0.82	−1.56 ± 0.87	−0.48 ± 0.49	3.71	0.04	8.90	0.01	2.97	0.08
	Post	0.03 ± 0.18	2.44 ± 0.17	0.58 ± 0.18	1.02 ± 0.1						
	Total	0.14 ± 0.45	1.15 ± 0.42	−0.49 ± 0.45	0.27 ± 0.25						
Cp1	Pre	0.19 ± 0.34	0.11 ± 0.32	−0.08 ± 0.34	0.07 ± 0.19	3.65	0.04	30.28	0.00	2.35	0.12
	Post	1.12 ± 0.28	2.35 ± 0.26	1.12 ± 0.28	1.53 ± 0.16						
	Total	0.65 ± 0.21	1.23 ± 0.19	0.52 ± 0.21	0.80 ± 0.12						

##### 3.2.3.1 Cz position

At the Cz position, a notable inter-group effect surfaced (*F* = 9.28, *p* < 0.05). The P300 amplitudes for the Sham Stimulation group (0.64 ± 0.22) and Transcranial Direct Current Stimulation (tDCS) group (0.87 ± 0.20) significantly surpassed those for the Tactile Gaming group (−0.35 ± 0.22). Simultaneously, we observed a significant intra-group effect, with pre-intervention P300 amplitudes (0.26 ± 0.27) being eclipsed by post-intervention amplitudes (1.03 ± 0.17, *F* = 11.88, *p* < 0.05). However, the interplay between inter-group and intra-group effects remained non-significant (*F* = 2.95, *p* > 0.05).

##### 3.2.3.2 Pz position

At the Pz position, noteworthy inter-group distinctions were evident (*F* = 6.19, *p* < 0.05). The tDCS group (1.06 ± 0.19) demonstrated markedly higher P300 amplitudes compared to the Tactile Gaming group (0.31 ± 0.20) and Sham Stimulation group (0.17 ± 0.20). Moreover, significant intra-group differences were noted at the Pz position, where pre-intervention P300 amplitudes (0.19 ± 0.19) lagged behind post-intervention amplitudes (0.83 ± 0.11, *F* = 9.09, *p* < 0.05). Nevertheless, the interaction between inter-group and intra-group effects did not reach significance (*F* = 3.46).

##### 3.2.3.3 FC2 position

At the FC2 position, a substantial inter-group effect emerged (*F* = 3.71, *p* < 0.05). The tDCS group (1.15 ± 0.42) exhibited elevated P300 amplitudes compared to the Sham Stimulation group (0.14 ± 0.45) and Tactile Gaming group (−0.49 ± 0.45). Similarly, a noteworthy intra-group effect was discerned, with post-intervention P300 amplitudes (1.02 ± 0.1) exceeding pre-intervention amplitudes (−0.48 ± 0.49, *F* = 8.09, *p* < 0.05). However, the interaction between inter-group and intra-group effects did not achieve significance (*F* = 2.97, *p* = 0.08 > 0.05).

##### 3.2.3.4 CP1 Position

At the CP1 position, a significant inter-group effect materialized (*F* = 3.65, p < 0.05). The tDCS group (1.23 ± 0.19) outperformed both the Sham Stimulation and Tactile Gaming groups. Furthermore, a significant intra-group effect was evident, with post-intervention P300 amplitudes (1.53 ± 0.16) surpassing pre-intervention amplitudes (0.07 ± 0.19, *F* = 30.28, p < 0.05). However, the interaction between inter-group and intra-group effects did not attain significance (*F* = 2.35, *p* = 0.12 > 0.05).

### 3.3 Frontal negativity (FRN) amplitude pre-and post-intervention in the IGT paradigm

A meticulous examination of Frontal Negativity (FRN) amplitude alterations across distinct electrode sites (Cz, Fz, FC2, FC1) was conducted, employing a rigorous repeated measures analysis of variance (ANOVA) in Table 3.

**Table 3:** Participants’ frontal negativity (FRN) amplitudes pre and post intervention (M ± SD).

Electrode	Phase	Group				Inter-Group		Intra-Group		Interaction effect	
		Pseudo-stimulation	tDCS	Exergame	Total	*F*	*P*	*F*	*P*	*F*	P
Cz	Pre	0.14 ± 0.42	0.52 ± 0.39	−1.36 ± 0.42	−0.24 ± 0.23	4.57	0.02	0.41	0.53	3.93	0.04
	Post	0.12 ± 0.29	−0.23 ± 0.27	0.00 ± 0.29	−0.04 ± 0.16						
	Total	0.13 ± 0.22	0.14 ± 0.21	−0.68 ± 0.22	−0.14 ± 0.13						
Fz	Pre	0.37 ± 0.25	−0.32 ± 0.23	−0.47 ± 0.25	−0.14 ± 0.14	5.41	0.01	1.11	0.30	2.73	0.09
	Post	−0.02 ± 0.21	−0.85 ± 0.19	−0.08 ± 0.21	−0.32 ± 0.12						
	Total	0.18 ± 0.17	−0.59 ± 0.16	−0.28 ± 0.17	−0.23 ± 0.1						
Fc2	Pre	0.08 ± 0.19	−0.17 ± 0.18	−0.16 ± 0.19	−0.08 ± 0.11	7.85	0.00	11.98	0.00	8.07	0.00
	Post	−0.53 ± 0.25	−1.56 ± 0.23	0.09 ± 0.25	−0.67 ± 0.14						
	Total	−0.22 ± 0.16	−0.87 ± 0.15	−0.04 ± 0.16	−0.38 ± 0.09						
Fc1	Pre	−0.01 ± 0.34	0.13 ± 0.32	−0.34 ± 0.34	−0.08 ± 0.19	0.21	0.81	0.00	0.97	0.64	0.54
	Post	−0.22 ± 0.25	−0.08 ± 0.24	0.04 ± 0.25	−0.09 ± 0.14						
	Total	−0.12 ± 0.21	0.03 ± 0.2	−0.15 ± 0.21	−0.08 ± 0.12						

#### 3.3.1 Cz position

At Cz, a noteworthy between-group effect surfaced (*F* = 4.57, *p* < 0.05). Specifically, the FRN amplitude within the tactile gaming group surpassed that of the transcranial direct current stimulation (tDCS) and sham stimulation groups significantly. Regrettably, there was an absence of a significant within-group effect. Furthermore, Cz revealed a substantial interaction effect between groups, elucidating discernible distinctions in FRN amplitude fluctuations among groups pre-and post-intervention.

#### 3.3.2 Fz position

Similarly, at Fz, considerable between-group differences materialized (*F* = 5.41, *p* < 0.05), with the tDCS group exhibiting elevated FRN amplitudes compared to the sham stimulation and tactile gaming groups. Unlike the observations at Cz, no significant within-group effect was noted at this electrode point, albeit post-intervention amplitudes registering lower values than their pre-intervention counterparts. Intriguingly, the interaction effect between groups, both within and between, failed to attain significance.

#### 3.3.3 FC2 position

At FC2, a significant between-group effect unfolded (*F* = 7.85, *p* < 0.05). The tDCS group conspicuously exhibited diminished FRN amplitudes in contrast to the sham stimulation and tactile gaming groups. Furthermore, a discernible within-group effect was noted, underscoring reduced FRN amplitudes post-intervention relative to pre-intervention values. Importantly, a notable interaction effect between groups came to light, indicating divergent responses in FRN amplitude fluctuations among groups pre-and post-intervention.

No substantial between-group or within-group effects were detected at FC1. Additionally, no significant interaction effect between groups, either within or between, manifested.

In summation, these findings accentuate the nuanced variations in FRN amplitudes across diverse electrode sites and the consequential impact of the intervention. Such results hold paramount importance in advancing our comprehension of FRN amplitude dynamics under disparate conditions and its intricate interplay with cognitive and neural mechanisms.

## 4 Discussion

This study employed diverse interventions to address smartphone addiction. Through a longitudinal tracking of smartphone addicts in different intervention conditions, subjective addiction levels, behavioral observations, and electroencephalographic (EEG) physiological data were collected. The results of our study showed that transcranial direct current stimulation (tDCS), exergames and pseudo-stimulation all exhibited significant therapeutic effects on smartphone addiction. Among these interventions, tDCS demonstrated particularly noteworthy regulatory effects on P300 wave amplitudes in the frontal, occipital, and central areas, as well as on the amplitudes of the central sulcus and frontal areas’ feedback-related negativity (FRN) waves. This suggests an augmentation of cognitive resources during decision-making tasks and an enhancement of inhibitory control in individuals with smartphone addiction. These findings contribute valuable insights into the neural mechanisms of smartphone addiction interventions, offering theoretical support for the development of relevant intervention strategies.

Furthermore, we directed our attention to the behavioral performance of smartphone addicts in a decision-making task (Iowa Gambling Task, IGT), an aspect less explored in prior studies on smartphone addiction (31). The IGT, known for eliciting emotional information through somatic marker signals, is associated with specific brain regions in the decision-making behavior of individuals with smartphone addiction (32), particularly the dorsolateral prefrontal cortex. Transcranial direct current stimulation (tDCS) interventions targeting the dorsolateral prefrontal cortex have been demonstrated to modulate the cognitive control circuit and enhance activity in this region (33) Additionally, researchers have found that bilateral dorsolateral prefrontal cortex stimulation (left cathode/right anode) at 2 mA effectively improves decision-making abilities and cognitive flexibility in individuals with gambling addiction (34). Consistent with these findings, both the tDCS intervention group and the exergame group in our study exhibited significantly better performance than the control group in the IGT task. This improvement may be associated with the direct modulation of decision-making-related brain regions.

In the fields of psychology and electrophysiology, the P300 amplitude is considered an indicator of the brain activity required for maintaining working memory, with its amplitude levels positively correlated with attentional resource allocation (35). As shown in Table 2, only the tDCS group’s participants, following intervention at the Cz, Fz, FC2, and FC1 electrode sites, exhibited significantly higher P300 amplitudes compared to pre-intervention, with significant differences observed (*p* < 0.05). This outcome was validated concerning subjective addiction severity scores and behavioral changes. Additionally, other studies indicate that tDCS in longitudinal interventions with alcohol-dependent and heroin-addicted patients modulates the disrupted state of neurotransmitters in this population (36), reduces drug cravings (37), and enhances executive control abilities (38). This may be attributed to the repetitive transcranial activation of the DLPFC by tDCS, leading to neuroplastic effects (39), promoting greater stability in neural connections, thereby facilitating positive behavioral transformations. Furthermore, the DLPFC plays a crucial role in executive control (40), and its neural circuit regulatory effects are anticipated to become a significant therapeutic approach in addiction recovery.

In contrast to P300 amplitude, Feedback-Related Negativity (FRN) represents a negative-polarity event-related potential (ERP) component elicited during feedback processing. A substantial body of empirical investigations has consistently substantiated that a core symptomatology of addiction resides in the aberrant processing of feedback. Individuals with addiction manifest heightened responsiveness to addiction-related reinforcement and associated cues relative to natural rewards (41). In the current investigation, individuals exhibiting smartphone addiction subjected to diverse intervention conditions, predominantly exhibited diminished amplitudes across CZ, Fz, FC2, and FC1 derivations, as delineated in Table 3. This underscores the positive impact of the intervention methodologies employed in this study on individuals characterized by elevated impulsivity and heightened sensitivity to smartphone-related stimuli.

In diverse studies, such as those examining internet addiction, distinct patterns have emerged. Notably, the experimental cohorts consistently exhibit significantly diminished Feedback-Related Negativity (FRN) amplitudes in comparison to their control counterparts (42). This observation is mirrored in individuals with gambling addiction, where FRN amplitudes are notably reduced during feedback associated with monetary stimuli in experimental paradigms. In contrast, Oberg’s investigation yielded incongruent findings, indicating an augmentation rather than a diminution in FRN amplitudes among participants with gambling addiction (43). Additionally, the examination conducted herein failed to discern gender disparities in smartphone addiction. Nevertheless, it is imperative to note that Andrade et al. (44) contend that females may manifest a heightened susceptibility to smartphone addiction, attributing this potential incongruity to the limited sample size. The paucity of research on smartphone addiction, especially in the realm of transcranial direct current stimulation and sensorimotor gaming, underscores the pressing need for comprehensive inquiries, bearing crucial practical implications in comprehending and mitigating the burgeoning problem of smartphone addiction. Noteworthy is the absence of untoward events or participant attrition during the intervention period, signifying the safety of both modalities for individuals grappling with smartphone addiction in their pursuit of recovery and restoration of health.

In contemplating future avenues of inquiry, we advocate for a nuanced exploration of the comorbid attributes between smartphone addiction and psychological symptoms to catalyze the development of precision-targeted intervention modalities. Building upon this foundation, it is advisable to deliberate upon the broadening of research cohorts and the implementation of methodological refinements, such as stratified randomization. These enhancements aim to fortify the external validity and generalizability of investigations. Their meticulous application is anticipated to intricately unravel the multifaceted and intricate interplay between smartphone addiction and psychological symptomatology, thereby furnishing robust underpinnings for the formulation of methodologically sound and academically rigorous treatment paradigms.

In summary, while this study has offered valuable insights into smartphone addiction intervention, certain limitations remain, particularly in terms of generalizability and sample representativeness. In addition, due to the issue of sample size, we did not conduct correlational analysis on the outcome indicators, which is also one of the limitations of our research. In future research, we plan to increase the sample size to analyze the correlations among changes in SAS-C scores, IGT performance, and ERP amplitudes more rigorously and objectively. Additionally, we anticipate that a deeper understanding of smartphone addiction will pave the way for the development of more effective intervention strategies to address the growing challenges associated with this issue.

## 5 Conclusion

All three interventions appeared to have alleviating effect on smartphone addiction. After 4 weeks, participants showed improved executive control and decision-making abilities. Specifically, significant effects were observed in the tDCS group, with increased P300 amplitudes in the frontal, parietal, and central regions, as well as FRN amplitudes in the central and frontal regions. This suggested that tDCS enhanced psychological resources and improved inhibition control capabilities.

## Data Availability

The raw data supporting the conclusions of this article will be made available by the authors, without undue reservation.
